# Editorial Notes

**Published:** 1903-06

**Authors:** 


### Editorial Notes.

**The XIVth International Congress of Medicine.**—Bristol was represented at this Congress by two members of the Editorial Committee of this *Journal*, and as delegates of the Bristol Medico-Chirurgical Society they had the honour of being received by His Majesty King Don Alphonso XIII. and his august mother. The Iberian peninsula is by no means a convenient region for an International meeting; but, nevertheless, the number of delegates and members inscribed at Madrid shows no indication of any diminution in the popularity of these triennial meetings.

For many years the reception of this Congress has indicated a growing rivalry between the different nations. We well remember the enthusiasm with which our Prince of Wales and the Crown Prince of Germany were received in St. James's Hall at the opening meeting of the great Congress in London in 1881; so it has been at each succeeding meeting; and it may be said of the Madrid meeting that the royal reception given by the King and the Queen mother were of the most cordial and flattering description. This has been perhaps the most striking feature of the meeting, for there has been no sensational scientific discovery announced. Some degree of sentiment seems to have attached itself to this meeting in

Spain: the question has frequently been asked, "Why go there to the land of toreadors and cavaliers, the land of ballads and romances, the land of castles and cathedrals, of fretted stone and age-toned pictures? What has it to do with microbes and antiseptics? And yet, the modern heroes of science have all been there, and have been fêted and honoured and presented at Court, and the queer world-jargon of many tongues proclaiming the new discoveries of medicine have sounded in the very halls where were once discussed the discoveries of one Columbus and the value of the Inquisition."[1] The historic city has justified its old reputation for courtesy and hospitality, and those who were present will have carried away with them many pleasant memories of the present age and many reminiscences of the glories of the past. Surely it is unnecessary to question the value to the world of meetings like this! Even if no new scientific progress appears to be made, the nations and the individual members cannot but profit greatly by such international conference and good fellowship.

Of the difficulties by the way—difficulties of language, difficulties of travel and such like,—many critics have written; but the well-organised party arranged by Mr. J. Y. W. MacAlister, on behalf of the Royal Medical and Chirurgical Society of London, gave its members every luxury with the minimum of the discomfort necessarily associated with long railway journeys. The Sud-express from Paris is a train which cannot well be surpassed, and beyond the frontier it should be remembered that an elevation of 2,000 feet is necessary to cross the Pyrenees, and that that elevation becomes over 5,000 feet in crossing the Sierra de Guadarrama, after which the railway drops to 2,400 feet at Madrid. As regards the language, it is not sufficiently realised that Spanish is perhaps the most widely-spoken next to the English, and that a little knowledge of the language is always to be desired by those who visit any foreign country.

On arrival in a new city on business of this kind, the first obvious duty of a delegate or member of Congress is to report himself at the official reception-room, and get his tickets and

[1] *Med. News*, 1903, lxxxii. 846.

programme of the proceedings. Here arose the first difficulty in Madrid; for on endeavouring to reach the spot arranged for the letter K, indicated by one's previously received ticket, it was found to be impossible to get there without a contest worthy of a game of football. On arrival there a very leisurely official, smoking his cigarette, was able to find your name after a prolonged search, and he then handed you the badge of the delegate and the two invitations of His Majesty the King. Other information would be forthcoming "mañana." There was no guide to the city, no official programme, no list of delegates or members, and no daily journal: all these duly appeared after many days—the guide-book as we were leaving Madrid, the list of members after we had left the city.

It was evident that the number in attendance was vastly in excess of the provision which had been made, and that the officials were not able to cope with the demands made upon them. Accordingly, it seemed impossible to learn when or where the opening meeting of the Congress was to be held; but with six or seven thousands of members and delegates it would not be easy to go wrong, and we found ourselves on Thursday, April 23rd, in the Teatro Real, in company with a large and distinguished party of British delegates, arrayed in Court and University robes. Both this meeting and the royal reception at the palace on the following day were brilliant successes, but they have been fully reported, and no further comment is needful. As regards the subsequent general meetings no information could be obtained, but we have since learned that Professor Arthur Thomson gave an address on "The Attitude of the Medical Profession toward Anthropology," and that several other general addresses were given in the amphitheatre of the Medical School. The sectional meetings were held in the Royal Library and Museum, a modern building, containing a large collection of modern pictures: the different galleries opening into each other were used for various sectional meetings, and hence the disturbance of the constant traffic, and the attractions of the magnificent pictures on the walls, must have seriously detracted from the utility of the work done by the sections.

The secretary announced at the opening meeting that of the total number of members enrolled, 6,961, about half of these, 3,530, were Spanish; of the remaining 3,431, Great Britain was represented by 238, whilst the United States of America had 195. The communications or papers announced for the meeting was 1,681. The English-speaking party was quite a representative one, including many well-known physicians and members of their families: they had also the pleasure of the company of the Rev. Canon Duckworth and the Rev. Sir Borradaile Savory, who were members of the section of hygiene.

As regards the city of Madrid, it is only just to remark that its buildings are quite metropolitan in character; the streets are wide, and the open spaces large: there is, however, one defect which could not escape observation: the rough granitic pitching ill-kept, and with frequent holes, is not worthy of a royal city of historic fame; it might have been supposed that the roads had been laid down by Phillip II., and had not received attention since his time. Madrid is above all things a city of picture galleries; the old masters of the Prado, and the modern tragedies of the Bibliotheca are collections of which any city might well be proud.

The pictures of Velasquez form a unique collection, but the Spanish Murillo is to be seen to better advantage in Seville, and the work of many of the old masters can be better seen in Florence, Munich, and other cities. Madrid is on the whole less attractive than most other large continental cities in museums and churches: its climate is not genial, although its elevation should make it worthy of the reputation of a European health resort: from the archæological standpoint it has no interest whatever.

Much has been said of the cherished national institution, the bull-fight: some of its critics write without knowledge, others fail to enter into its spirit from the Spanish point of view. It is true that there is little sport in the game; the matadors run very little risk, and the blind-folded horses are entirely at the mercy of the enemy: it must not be forgotten that the bulls and the horses are there to be killed, that the bulls are fine Andalusian animals bred for the purpose, and that the horses are worn out and useless. It is not a humane kind of death either for horse or bull, but the rules of the game are rigidly observed, and the animals are not tortured indefinitely. And yet to the English mind such exhibitions cannot but appear to be brutalising in their tendency, and it is surprising they should excite such wild enthusiasm even in a highly excitable race. The fêtes are held in every large city of Spain; they are of great antiquity, dating from the time of the Moors, or even a long way further back to the time of the amphitheatres of the Roman Empire; they are patronised by all classes of society from the highest to the lowest, and it would be as impossible a task to suppress them as to prevent an English schoolboy from playing cricket in the summer term.

A visit to the far-famed imperial city of Tolédo is inevitable to anyone so near to it as Madrid, and "very solemn and impressive does Tolédo seem to the arriving traveller, with its grey old bridge, its massive walls, its solid-looking buildings rising one above another crowned by the Alcazar, its feet for ever washed by the Tagus, which here makes a loop round the ancient city. Rocky and sandy hills of indescribably barren aspect rise on the other side of the drab-coloured waters, and nowhere is this picture of decay and death relieved by a warmer tint. The intense gloom of this once mighty town fascinated me strangely, and I lingered there day after day till its stately air of majestic resignation was indelibly fixed on my mind. It is a wonderful place. I grew at last to think it the most wonderful place I had ever seen"[1]. The beauties of the Cathedral, with its five naves and their eighty-eight columns, the grand Moorish gateway, called Puertâ del Sol, the Moorish mosque, now called the Church of El Christo de la Luz, and the many historic reminiscences of the Moorish occupation cannot here be described.

Another day excursion from Madrid is that to the Escorial, where the kings and queens of Spain find their last resting-place, in the Panteon, an octagonal chamber immediately underneath the high altar of the church above, and where everything reminds one of Phillip II., our own Queen Mary's husband.

[1] Mrs. Main, *Cities and Sights of Spain*, p. 114.

What can be said of Granada and the Alhambra, which has not been said many times before? It is a weary pilgrimage to Seville, Cordova, Granada, and the other cities of the South; but no one can pretend to a good knowledge of Spain who has only visited the bare granitic wastes of the North. Trains are infrequent and slow, and the way is long, but the recompense is great. Hare (*Wanderings in Spain*) remarks: "There is nothing more interesting than the awakening in a place new, and yet so old, so well-known from stories and pictures of earliest childhood, as Granada. It was like an awakening in Paradise." The whole Alhambra teams with reminiscences of the romantic history of the Moors, terminating with the surrender of the city by Boabdil, in 1492, when the long duel between Spaniard and Moor came finally to an end, and the defeat of the Goths was avenged; in this year also occurred the discovery of America, and Spain first took a leading place amongst the Powers of Europe.

**Winsley Sanatorium: Laying the Foundation Stone.**—The laying of the Foundation Stone of this Sanatorium by Lady Dickson-Poynder marks the progress of the scheme, and the large and representative company which assembled on the site to do honour to the occasion and to Lady Dickson-Poynder shows how widespread is the interest in this prospective Home.

The CHAIRMAN (Sir John Dickson-Poynder) having given a short and interesting account of the work,

Dr. WEATHERLY, the Chairman of the Executive Committee, stated that on March 26th, 1903, they had collected altogether £9,758—paid and promised. Of that they had spent on the purchase of the land, on levelling the site, on planting trees, making roadways, legal expenses, office expenses, advertising and printing, and holding public meetings throughout the three counties, £3,918, leaving a balance of £5,840. Since March 26th up to the 3rd of June they had been paid or promised £1,000, increasing the balance to £6,840. The contract for the present building was £6,877; the furniture and electric light would cost about £1,450, and if they built a separate laundry, which was almost a *sine quâ non*, it would cost another £1,000

which brought the sum required for the building as at present arranged to £9,327, so that for the present undertaking they required a further sum of £2,587. It was proposed to provide their beds as they obtained funds, instead of waiting for funds for a big extension, in blocks, each of which would receive about ten beds and cost about £1,400. So to complete the whole scheme and give accommodation for sixty patients they wanted about £9,000. With regard to the beds in the building now commenced, they would try to keep one-third of them for free nominations, and these would be available for persons at a charge not exceeding 10s. a week. In that building they hoped to have six such beds, which meant that the Committee would have to provide, to maintain all the patients in that building, not more than £250 a year in subscriptions from the three counties —Somerset, Wilts, and Gloucestershire—and the City of Bristol. Beds would be maintained as follows: By the City of Bristol, 4; Bath Corporation, 2; Swindon Corporation, 2; Gloucester Corporation, 1; Messrs. Fry and Sons, Bristol, 1; Mr. J. B. Clark, of Street, 1; Mr. and Mrs. W. S. Clark, of Street, 1. The G.W.R. employeés at Swindon had already paid for the building of a bed, and hoped to maintain it; the Dean Testimonial Fund at Swindon had provided for the building of a bed, and meant to maintain one, and the Committee controlling the memorial to Mr. Dean were providing a liege-halle, or rest pavilion, in which four or five patients could lie down. Trowbridge was also collecting subscriptions for the purpose of building and maintaining a bed, which meant that fifteen beds would be endowed.

Lady Dickson-Poynder, amid applause, received a beautiful bouquet from Miss Watson Williams, daughter of the hon. general treasurer (Dr. Watson Williams, of Clifton), and granddaughter of the late Dr. Long Fox, who was practically the founder of this branch of the association.

The short religious service then commenced, Prebendary Boyd (Rector of Bath), saying prayers, the responses being by the Abbey choir, who sung the following hymn, which had been especially written for the occasion by Mr. Fred E. Weatherly.

Be with us Father, where we meet,
Bless what we do this day!
Our work begun—that work complete
And guide it in Thy way.

O, keep the sufferers in Thy care
Who seek this sheltering place;
O, heal them with Thy bounteous air,
Refresh them with Thy grace.

O, bless the loving hands that tend
The busy brains that plan
To use the mercies Thou dost send
For help of suffering man.

And bless us also, God most high,
And keep us in Thy ken;
Be with us now, and when we die
O, Lord, be with us then!

Lady Dickson-Poynder then laid the stone. Her ladyship used for the spreading of the mortar a beautiful silver trowel with ivory handle, which was handed to her by Dr. Weatherly.

The Bishop of Bristol then offered an appropriate prayer and pronounced the benediction.

Dr. A. Hillier, having spoken of the suitable nature of the site for the Sanatorium and the loveliness of the surrounding scenery, congratulated most heartily those who were associated with the movement upon the work that had been done so far. He had no doubt that in the future they would have equal success, and carry the scheme to a successful termination. They were to be congratulated, not only as representing the three counties named, but because they were setting an example to the whole country. In Germany there were at present 7,000 beds at the disposal of the working classes who might be suffering from consumption; yet in England and Wales there were not at present in proper sanatoria more than as many hundreds of beds for that purpose. They were, therefore, in that matter doing a great and much needed national work.

We gladly offer our cordial congratulations to the Executive Committee on their increased activity, and on the increasing interest manifested in this scheme, which promises to be a success in the near future. Meanwhile, it is clear that the funds are not adequate for the work which has to be done, and that the Committee can scarcely be expected to make up the deficiency without greatly increased assistance from those who are convinced of the urgent necessity for the work.

### Notes on Preparations for the Sick.

